# Carbonization of Graphene-Doped Isocyanate-Based Polyimide Foams to Achieve Carbon Foams with Excellent Electromagnetic Interference Shielding Performance

**DOI:** 10.3390/ma14247551

**Published:** 2021-12-09

**Authors:** Hui Jing, Zongnan Miao, Zhong Zeng, Hui Liu, Shengtai Zhou, Huawei Zou, Mei Liang

**Affiliations:** 1 The State Key Laboratory of Polymer Materials Engineering, Polymer Research Institute of Sichuan University, Chengdu 610065, China; 15822218752@163.com (H.J.); miaozonnan@163.com (Z.M.); liangmeiww@163.com (M.L.); 2 Safety Environment Quality Surveillance and Inspection Research Institute of CNPC Chuanqing Drilling & Exploration Corporation, Chengdu 618300, China; ktzengz_sc@cnpc.com.cn (Z.Z.); Liuh_kt@cnpc.com.cn (H.L.)

**Keywords:** polyimide, carbon foam, graphene, electromagnetic interference shielding, carbonization temperature

## Abstract

Lightweight carbon foams with excellent electromagnetic interference (EMI) shielding performance were prepared by carbonization process, using isocyanate-based polyimide foams as carbon precursors. The influence of carbonization temperature and graphene-doping on the morphological, electrical and EMI shielding effectiveness (SE) of corresponding carbon foams was studied in detail. Results showed that the addition of graphene was beneficial to the improvement of electrical conductivity and EMI shielding performance of carbon foams. The electrical conductivity of carbon foams increased with the carbonization temperature which was related to the increase of graphitization degree. Collapse of foam cells was observed at higher carbonization temperatures, which was detrimental to the overall EMI SE. The optimal carbonization temperature was found at 1100 °C and the carbon foams obtained from 0.5 wt% graphene-doped foams exhibited a specific EMI SE of 2886 dB/(g/cm^3^), which shows potential applications in fields such as aerospace, aeronautics and electronics.

## 1. Introduction

Nowadays, electromagnetic interference (EMI) is becoming a severe problem with the large-scale utilization of electronic devices in telecommunication and electrical industries among others, especially in the wake of 5G/6G era [1,2]. The emitted electromagnetic waves from these electronic devices are likely to cause malfunction of precision devices and disrupt the biological systems of human bodies [3,4,5]. Conventionally, metals are considered as excellent EMI shielding materials due to their intrinsically high electrical conductivity. However, they suffer drawbacks such as high density, poor resistance to corrosion and oxidation, high cost and complex processing steps, which severely limit their application in industrial sectors [6,7,8].

Conductive polymer composites (CPCs) bear the merits of tailorable electrical conductivity (depending on the concentration of conductive fillers), lightweight, fast processing and excellent resistance to corrosive conditions. It has been the core pursuit of using CPCs to replace metallic parts in order to meet the requirements of weight reduction and fast production [9,10]. Normally, polymer composites would become conductive if sufficient conductive fillers are added to build sufficient conductive pathways within the host matrix [11,12]. The commonly adopted fillers include carbonaceous fillers such as graphite, carbon nanotubes, graphene, and metal particles/nanowires such as copper powders and silver nanowires [13,14,15]. Graphene, which is termed as a thin layer of sp^2^-bonded carbon atoms, has been considered as a promising additive to endow EMI shielding properties to polymer composites thanks to its intrinsically high electrical conductivity [16,17,18,19]. Therefore, great effort has been devoted to fabricating graphene-containing polymer composites with excellent EMI shielding properties [20,21,22].

Recently, developing porous CPCs with excellent EMI shielding effectiveness is gaining attention due to their low density, improved material efficiency and high specific EMI shielding effectiveness (SE), which demonstrates potential application in the fields of aerospace and aeronautics, automobiles and electronics [23,24,25,26,27]. For example, Chen et al. [28] prepared a porous dual-continuous double percolated polystyrene (PS)/polymethyl methacrylate (PMMA)/carbon nanotubes (CNT) composites by combining melt blending and supercritical carbon dioxide foaming process. It was found that the percolation threshold was reduced from 0.18 to 0.14 vol%, and the specific EMI SE was increased from 37.79 to 57.70 dB/(g/cm^3^) when compared the foamed samples with their solid counterparts. Zeng et al. [29] reported that the specific EMI SE of porous water-borne polyurethane/CNT composites reached as high as 1148 dB/(g/cm^3^) in the X-band which was related to the typical porous structure that formed by freeze-drying method and electrical conductivity of the foam cell walls. Wang et al. [26] fabricated porous poly(vinylidene fluoride) (PVDF)/CNT composites by hot pressing and selective etching method. They reported that the EMI SE of PVDF/CNT 15 wt% nanocomposites reached as high as 56.72 dB (density: 0.79 g/cm^3^, thickness: 2 mm). The other approach is adopting the carbonization process to prepare electrically conductive carbon foams using organic foams as precursors, which can simultaneously provide lightweight and exceptional EMI shielding performance. Gu et al. [30] prepared MXene-containing carbon foams with the aid of vacuum assisted impregnation of MXene, followed by freeze-drying method. Results showed that the carbon foams with 8.5 wt% MXene exhibited a specific EMI SE of 216.9 dB/(g/cm^3^), coupling with excellent flame retardancy and heat insulation properties. Kumar et al. [31] reported that carbon–cenosphere composite foams were prepared by carbonizing of polyurethane foams impregnated with phenolic resin and cenospheres at 1000 °C. The EMI SE of carbon foams with 30 wt% cenospheres was 48.6 dB which was 92.9% higher than that of unfilled counterparts. Li and co-workers [32] prepared polyimide (PI) derived carbon foams and they reported an EMI SE as high as 41.1 dB with the introduction of adenine in the molecular chain of PMDA-ODA.

Conventionally, carbon foams were prepared as per the following methods, which include blowing of carbon precursors (i.e., pitch, phenol-formaldehyde resin), template carbonization, compression of exfoliated graphite, assembly of graphene nanosheets, and the other methods (such as the carbonization of mixtures of either hollow phenolic spheres or hollow carbon spheres with furfuryl alcohol) [33,34]. In addition to the above-mentioned approaches, PIs are commonly employed as the precursors for preparing carbon materials due to their high carbon yield and flexible adjustability of molecular chains [35]. Inagaki et al. [35] reported that little cracks were generated when carbonizing PI films at high temperatures. Thus, it is believed that PI foams can be considered as a good candidate for preparing the carbon foams. The methods for preparing PI foams include polyamic acid foaming, polyester ammonium salt precursor foaming, microsphere foaming and isocyanate-based foaming method [36]. The isocyanate-based foaming method is simple and easily implemented since it uses carbon dioxide that produced in situ as the foaming agent [37,38].

In the present study, isocyanate-based foaming method was employed to prepare PI foams which were used as the precursors to prepare carbon foams via carbonization. Graphene was incorporated as a functional filler to improve the electrical and EMI shielding performance of carbon foams. This study focused on the influence of carbonization temperature and graphene-doping on the morphological, electrical, and EMI shielding properties of carbon foams. It was found that the optimal EMI SE was achieved for 0.5GR doped PI foams when carbonized at 1100 °C. This study provided a viable route for preparing carbon foams with excellent EMI shielding performance, which show potential applications in the fields of aerospace, aeronautics, and electronics among others.

## 2. Experimental

### 2.1. Materials

Pyromellitic dianhydride (PMDA) was purchased from Aladdin (Shanghai, China). Polymethylenepolyphenyl isocyanate, PM200, with a -NCO content of 29~32% was supplied by Wanhua Chemical Group (Yantai, China). Dimethylacetamide (DMAc) and methanol was purchased from Chengdu Kelong Chemical Company (Chengdu, China). Homemade distilled water was used as foaming agent. Graphene (GR) particles with prepared in our laboratory as per [39,40]. According to Zhang et al. [40], the average particle size was between 1.5 and 20 μm with a thickness of 1.1 nm.

### 2.2. Preparation of Isocyanate-Based Polyimide Foams and Their Derived Carbon Foams

Prior to use, PMDA was dried thoroughly at 150 °C to remove eventual traces of moisture. The isocyanate-based polyimide (PI) foams were prepared as follows. Firstly, PMDA, CH_3_OH and DMAc were charged into a plastic beaker with extensive stirring. Afterwards, distilled H_2_O was added to the above mixture with stirring to yield white-color slurry. Then, GR and PM-200 were simultaneously added to the above mixture with aid of external stirring. Subsequently, they were poured into the mold to obtain PI intermediate foams by free-rising method. Finally, PI foams were obtained after thermal imidization at 250 °C. The doping content of GR was fixed at 0.5 wt%. Afterwards, the carbon foams (CFs) were obtained after carbonization process at different temperatures. For example, pure PI foams that carbonized at 800 °C were denoted as CF-800a whereas samples that carbonized from 0.5GR doped samples were designated as CF-800b. Such nomenclature was applicable to the other sample systems.

### 2.3. Characterization

#### 2.3.1. Scanning Electron Microscopy

All samples were gold sputtered and images were taken using a JEOL JSM-9600scanning electron microscope (SEM, JEOL, Tokyo, Japan) at the operating voltage of 15 kV.

#### 2.3.2. Apparent Density

The apparent density of foams was determined as per GB/T 6343-2009. Briefly, samples with dimensions of 30 × 30 × 30 mm^3^ were cut from the obtained foams. The mass of each sample was weighed using a precision scale. The apparent density of foams was determined by mass divided by volume. Five replicates were measured for each sample.

#### 2.3.3. X-ray Diffraction

X-ray diffraction scan for each sample was carried out on an X-ray diffractometer (XRD, Ultima IV, Rigaku, Tokyo, Japan) with Cu-Kα radiation (K = 0.154 nm, where K is the wavelength of X-ray).

#### 2.3.4. Electrical Conductivity

The resistance of the carbonized foams (CFs) was determined using Agilent 34401A digital electrometer (Agilent Technologies Inc., Santa Clara, CA, USA). The electrical conductivity of CFs was calculated as per the following equation:

$$\sigma =\frac{1}{\rho }=\frac{d}{RS}$$

where *σ* is the electrical conductivity, *ρ* is the resistivity, *d* is the thickness between the copper electrodes. *R* is the resistance and *S* is the contact area between sample and the copper electrode.

#### 2.3.5. Electromagnetic Interference Shielding Effectiveness

The EMI shielding effectiveness (EMI SE) of each sample was tested in a frequency range from 8.2 to 12.4 GHz using an Agilent N5247A vector network analyzer (Agilent Technologies Inc., Santa Clara, CA, USA). The Agilent vector network analyzer was calibrated using standard APC-7 connector open, short, and 50  Ω loads. Samples with a diameter of 12  mm were placed in the sample holder and connected through Agilent 85132F coaxial line to separate VNA ports. Samples with a thickness of 2  mm were prepared for testing.

## 3. Results and Discussion

### 3.1. Morphology

**Figure 1:**
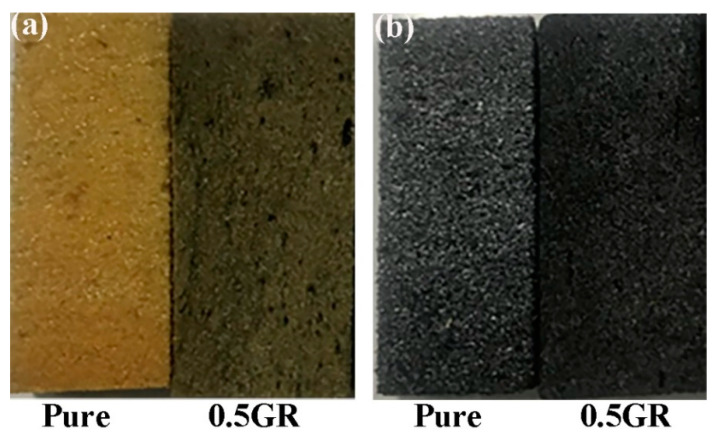
The morphology of pure and graphene-doped PI foams (**a**) before and (**b**) after carbonization at 1200 °C.

**Figure 2:**
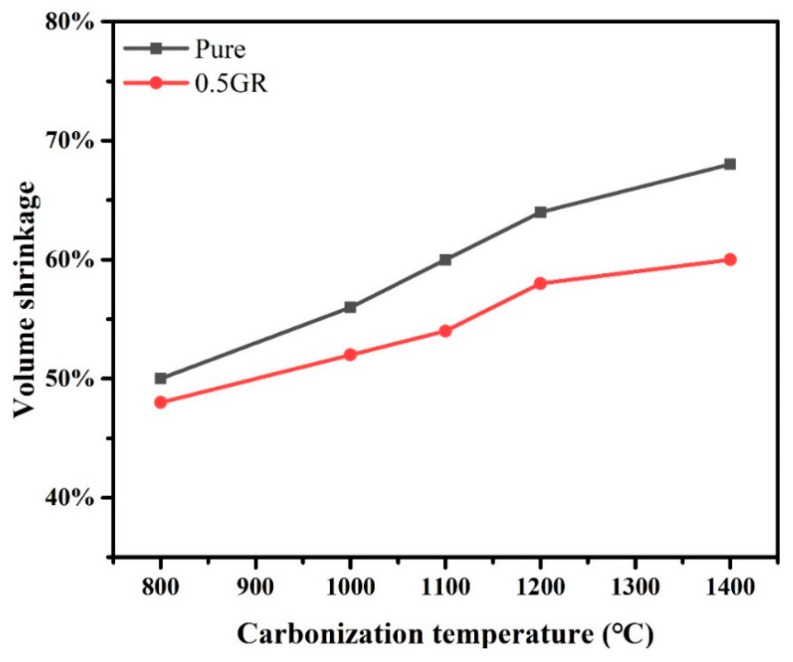
The volume shrinkage of pure PI and 0.5GR doped PI foams at different carbonization temperatures.

Figure 1 shows the optical images of pure and graphene-doped PI foams before and after carbonization at 1200 °C. It is clear that the appearance of carbonized foams became darker after carbonization treatment, which could be attributed to the pyrolysis of PI polymer chains at high temperature, thereby leading to the significant volume shrinkage of PI foams, as displayed in Figure 2. In addition, the volume shrinkage increased with increasing carbonized temperatures, where samples carbonized at 1400 °C exhibited the highest shrinkage for both pure PI and 0.5GR doped PI foams. According to literature [35,41], such shrinkage was mainly related to the generation of volatile gases such as CO, N_2_ and CH_4_ during the pyrolysis of PI foams. Impressively, the carbonized foams still maintained intact after carbonized at 1400 °C, which suggested the excellent thermal stability of the carbon foams. Figure 2 showed that the volume shrinkage of 0.5GR doped PI foams was lower than their pure PI counterparts, especially for the foams that carbonized at 1400 °C. This indicated that the addition of graphene particles was beneficial to enhance the mechanical properties of the skeleton of PI foams, which could withstand the internal stress that generated during the pyrolysis process, thereby demonstrating a much lower shrinkage when compared with pure PI samples [42].

**Figure 3:**
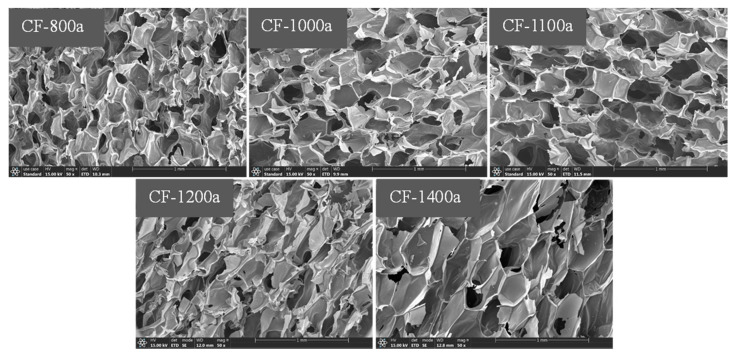
SEM images of pure PI foams that carbonized at 800 °C, 1000 °C, 1100 °C, 1200 °C and 1400 °C, respectively.

**Figure 4:**
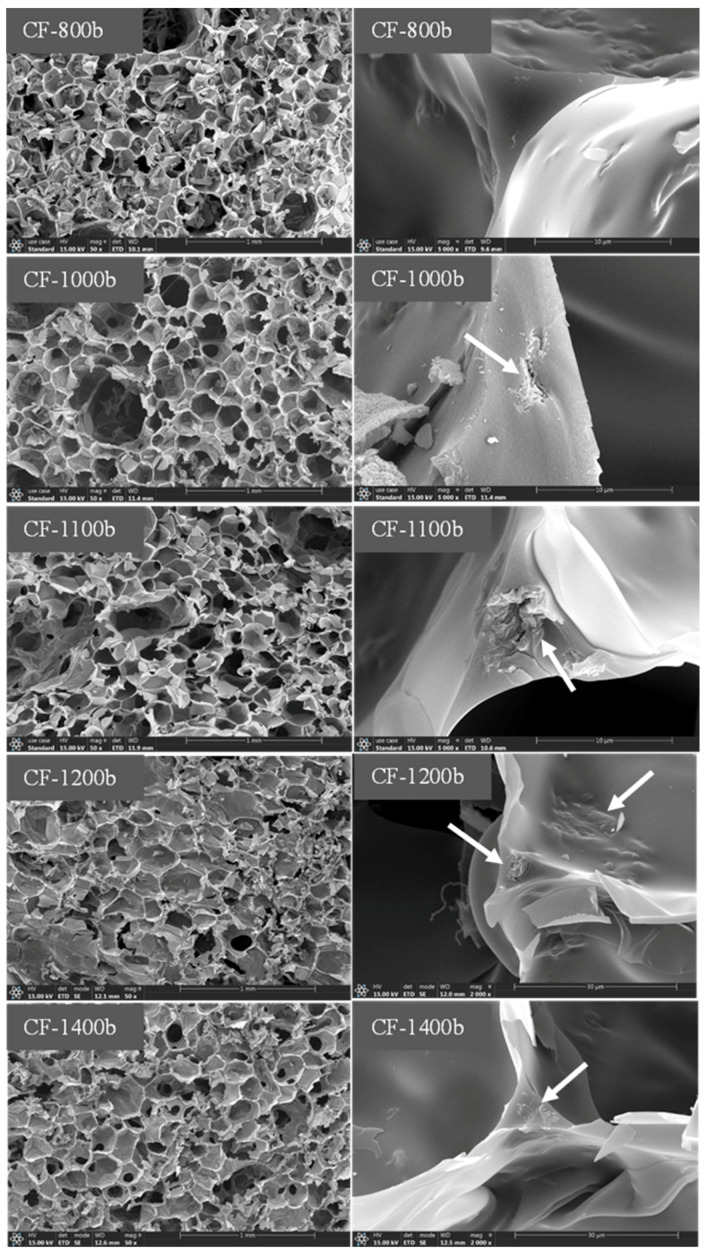
SEM images of 0.5GR doped PI foams that carbonized at 800 °C, 1000 °C, 1100 °C, 1200 °C and 1400 °C, respectively. The presence of GR was labeled using white arrows.

The microstructure of pure PI foams and 0.5GR doped PI foams that carbonized at different temperatures is displayed in Figure 3 and Figure 4, respectively. Results showed that the cell size of carbon foams was smaller than that of original PI foams. In addition, collapse of foam cells was observed in the carbon foams, especially for 0.5GR doped carbon foams, which was likely related to the pyrolysis induced coalescence of foam cells. However, the cell size of carbon foams that derived from pure PI foams increased slightly when the carbonization temperature was higher than 1200 °C. This was believed to be related to the release of volatile gases that formed during carbonization process which contributed to the slight increase of foam cell sizes. Furthermore, traces of graphene particles (as pointed out using white arrows) were detected in the triangle region that formed among foam cells, as displayed in the high-resolution SEM images of Figure 4.

### 3.2. Apparent Density and Raman Analysis

**Figure 5:**
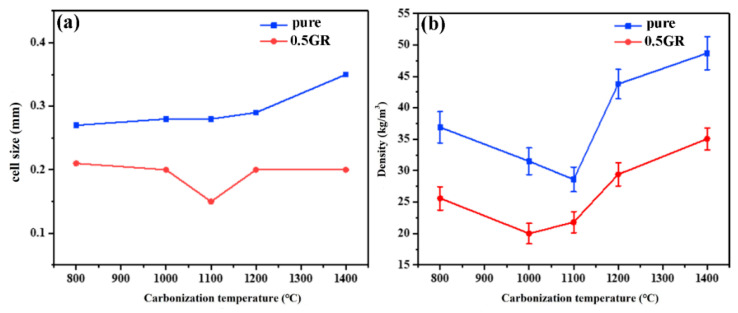
The (**a**) average cell size and (**b**) apparent density of carbonized pure and 0.5GR doped PI foams as a function of temperature.

The average cell size and apparent density of carbonized pure and 0.5GR doped PI foams are displayed in Figure 5a,b, respectively. Figure 5a shows that the average cell size of pure PI foams increased slightly with increasing carbonization temperature, which suggested that the cell size increased with the release of volatile content at higher carbonization temperatures [35]. However, there was no appreciable increase of foam cell size for 0.5GR doped PI foams which suggested that the presence of GR increased the strength of foam cells, thereby withholding the increase of foam cells size during carbonization process. In general, the average cell size of 0.5GR doped carbon foams was smaller than that of pure carbon foams. In this scenario, the added GR particles acted as the nucleating agents which led to the formation of a larger number of cells, thereby resulting in the decrease of cell sizes [43]. It is known that the apparent density of carbon foams was determined by the doping content of GR particles and carbonization process. Figure 5b shows that the apparent density of pure PI foams derived carbon foams was higher than that of 0.5GR doped counterparts, which was related to the higher volume shrinkage of pure PI foams than 0.5GR doped counterparts [44]. The apparent density of pure PI and 0.5GR doped PI foams reached 37 and 26 kg/m^3^ when the carbonization temperature was 800 °C. Moreover, there was an incremental decrease of foam density with increasing temperature up to 1100 °C. In this scenario, it was induced that the rate of mass loss was higher than that of the volume shrinkage during the thermal treatment process. After 1100 °C, the volatile organic content was nearly fully carbonized and the mass loss was negligible; however, the carbonized foams experienced a further graphitization process which might lead to a slight reduction in the foam size, thereby contributing to an increase of apparent foam density [45].

**Figure 6:**
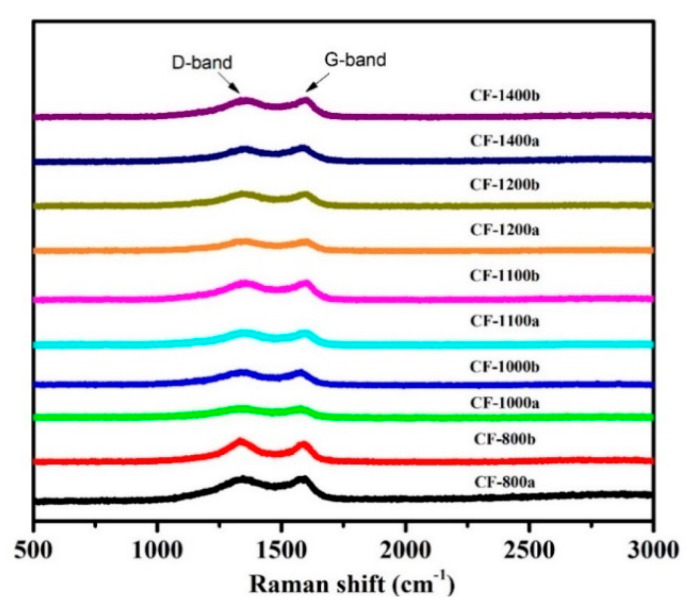
The Raman spectra of carbonized pure and 0.5GR doped PI foams at different temperatures.

The Raman spectra of carbon foams that carbonized at different temperatures are given in Figure 6. According to literature [46,47,48], the prominent peak that detected at 1350 cm^−1^ was designated to the D-band which was related to the presence of disorders or defects in carbon structure. The peak that observed in the vicinity of 1580 cm^−1^ was related to the crystalline phase in carbon structure. The value of I_D_/I_G_ was often used as an indicator to assess the degree of graphitization in carbon materials [49]. A reduction in the values of I_D_/I_G_ was related to the increasing graphitization of carbon materials after thermal treatment. The values of ID/IG of carbonized pure PI and 0.5GR doped PI foams at different temperatures are tabulated in Table 1. It was clear that the values of ID/IG for carbonized pure PI foams was reduced from 1.588 (carbonized at 800 °C) to 1.180 (carbonized at 1400 °C); the values of ID/IG for carbonized 0.5GR doped PI foams was reduced from 1.417 (carbonized at 800 °C) to 1.183 (carbonized at 1400 °C). This indicated that the carbonization at higher temperatures was favorable for the formation of crystalline graphite phase, which was consistent with the following XRD results.

### 3.3. XRD

**Figure 7:**
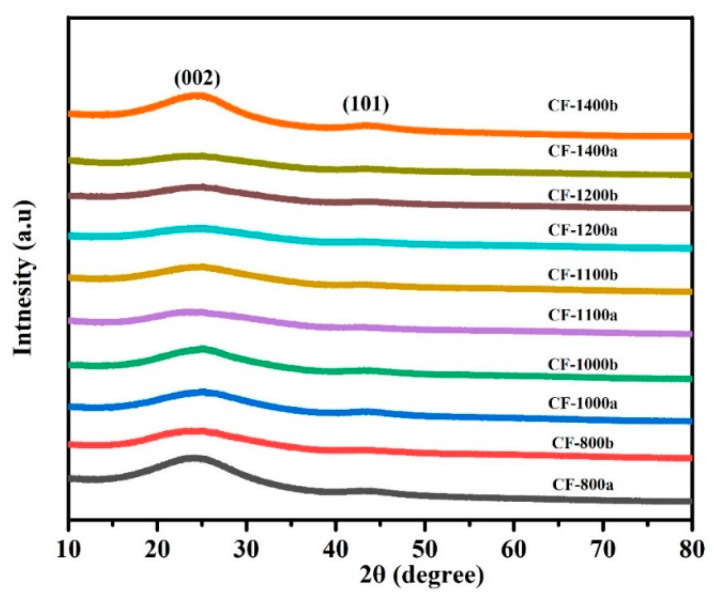
The XRD spectra of carbonized pure and 0.5GR doped PI foams at different temperatures.

The XRD spectra of carbon foams that formed at different carbonization temperatures are displayed in Figure 7. Basically, disordered carbon would be generated when PI foams were pyrolyzed at 600 °C. The disordered carbon would be transformed to form crystalline structure with increasing carbonization temperature, suggesting a transition from the amorphous state to crystalline form due to an increase of graphitization degree [50,51]. Figure 7 showed that the (002) peak of carbon foams increased from 24.3°to 25.06°. In addition, the (101) peak appeared when the carbonization temperature was higher than 1100 °C, which indicated that the graphitization content was enhanced after thermal treatment at higher carbonization temperatures.

### 3.4. Electrical Conductivity

**Figure 8:**
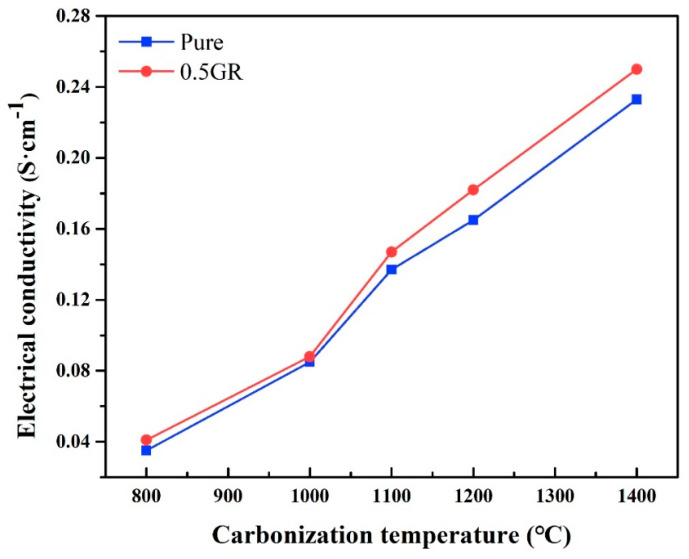
The electrical conductivity of pure PI and 0.5GR doped PI foams as a function of carbonized temperature.

The electrical conductivity (*σ*) of pure and 0.5GR doped PI foams that carbonized at different temperatures is shown in Figure 8. Results showed that the *σ* of carbon foams increased with increasing carbonization temperature, with samples carbonized at 1400 °C displayed the highest values of *σ*. Such phenomenon was undoubtedly related to the increase of graphitization content of carbon foams with increasing treatment temperatures, thereby leading to an increase of *σ*. In addition, the samples with 0.5GR exhibited higher *σ* when compared with the pure PI foam derivatives, which was related to the intrinsically higher *σ* of graphene and the added graphene acted as function fillers that contributed to a slight increase of *σ*.

### 3.5. EMI Shielding Effectiveness

**Figure 9:**
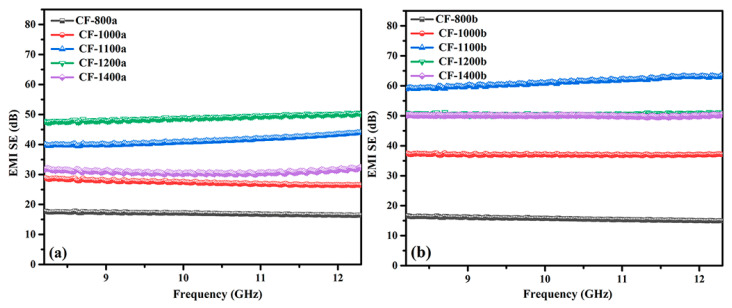
Electromagnetic shielding effectiveness of carbonized (**a**) pure PI foams and (**b**) 0.5GR doped PI foams at different temperatures.

The electromagnetic shielding effectiveness (EMI SE) of carbon foams that formed at different temperatures is displayed in Figure 9. Unlike metallic parts, the EMI SE of carbon foams was mainly comprised of reflection loss and absorption loss due to their typical foam structure [52]. The EMI SE of both pure PI foam and 0.5GR doped PI foams was relatively low when the carbonization temperature was merely 800 °C. As reported previously, the graphitization content and *σ* of carbon foams increased with increasing treatment temperatures, which was advantageous to the increase of EMI SE. However, the EMI SE of carbon foams that derived from pure PI foams decreased when the carbonization temperature increased to 1400 °C whereas such phenomenon was detected for 0.5GR doped foams when the carbonization temperature was above 1200 °C. Collapse of foam cells might have occurred when carbonization temperature was extremely high, and the formation of large size defects led to the penetration or direct transmission of electromagnetic waves. This was exactly the case for 0.5GR doped PI foams, as reported in Figure 3. However, the EMI SE of 0.5GR doped carbon foams was still higher than that of pure PI foam derived carbon foams, which was attributed to the difference in *σ* (see Figure 8). The optimal EMI SE was achieved for 0.5GR doped PI foams when they were carbonized at 1100 °C.

**Figure 10:**
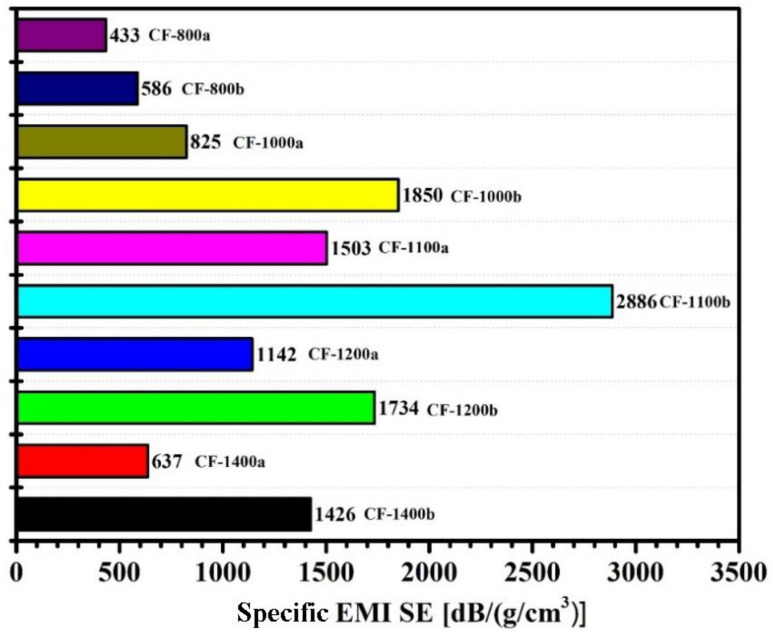
Electromagnetic shielding effectiveness per unit density of carbonized pure PI and 0.5GR doped PI foams at different temperatures.

The specific EMI SE, that is EMI SE per unit density [19], of the obtained carbon foams is displayed in Figure 10. Results indicated that the carbonized 0.5GR doped PI foams performed better when compared with that of pure PI foam derivatives. The values of specific EMI SE were lowest for both foams when the carbonization temperature was 800 °C, which was related to the lower *σ* of corresponding foams. The *σ* of carbon foams increased with further increasing carbonization temperature and the reduction of foam density in the vicinity of 1100 °C contributed to the increase of specific EMI SE for subsequent foams. For example, the specific EMI SE of pure PI foams that carbonized at 1100 °C reached 1503 dB/(g/cm^3^) and the highest value of specific EMI SE of 0.5GR doped PI foams that carbonized at 1100 °C was 2886 dB/(g/cm^3^). The specific EMI SE decreased with further increasing carbonization temperature, which was related to the following factors: (1) the foam density increased with increasing treatment temperature; (2) more defects of carbon foams would be generated at higher treatment temperatures. Both factors contributed to a decrease of specific EMI SE of resultant carbon foams.

## 4. Conclusions

In this work, isocyanate-based polyimide (PI) foams which were prepared by free-rise foaming method were employed as the carbon precursors aiming to prepare carbon foams with lightweight and excellent EMI shielding performance. Graphene particles were doped to enhance the electrical conductivity of PI foams. The effect of carbonization temperature on the electrical conductivity, morphological and EMI SE was detailed. Results showed that the electrical conductivity of carbon foams increased with carbonization temperature which was attributed to the increase of graphitization degree of the carbon foams, further supported by Raman and XRD measurements. The optimal carbonization temperature was 1100 °C and the PI/0.5GR derived carbon foams showed a specific EMI SE up to 2886 dB/(g/cm^3^), which is attractive for applications in high-end engineering fields such as aerospace, automobiles and electronics.

## Figures and Tables

**Table 1:** The values of I_D_/I_G_ of carbonized pure and 0.5GR doped PI foams at different temperatures.

Sample	I_D_/I_G_	Sample	I_D_/I_G_
800 °C pure	1.588	1100 °C GR	1.280
800 °C GR	1.417	1200 °C pure	1.284
1000 °C pure	1.358	1200 °C GR	1.249
1000 °C GR	1.282	1400 °C pure	1.180
1100 °C pure	1.231	1400 °C GR	1.183

## Data Availability

The research data are available from corresponding authors upon reasonable request.
